# Anti-S1 MERS-COV IgY Specific Antibodies Decreases Lung Inflammation and Viral Antigen Positive Cells in the Human Transgenic Mouse Model

**DOI:** 10.3390/vaccines8040634

**Published:** 2020-11-01

**Authors:** Aymn T. Abbas, Sherif A. El-Kafrawy, Sayed Sartaj Sohrab, Ashraf A. Tabll, Ahmed M. Hassan, Naoko Iwata-Yoshikawa, Noriyo Nagata, Esam I. Azhar

**Affiliations:** 1 Special Infectious Agents Unit, King Fahd Medical Research Center, King Abdulaziz University, Jeddah 21589, Saudi Arabia; saelkfrawy@kau.edu.sa (S.A.E.-K.); ssohrab@kau.edu.sa (S.S.S.); hmsahmed@kau.edu.sa (A.M.H.); eazhar@kau.edu.sa (E.I.A.); 2 Department of Medical Laboratory Sciences, Faculty of Applied Medical Sciences, King Abdulaziz University, Jeddah 21589, Saudi Arabia; 3 Biotechnology Research Laboratories, Gastroenterology, Surgery Centre, Mansoura University, Mansoura 35511, Egypt; 4 Department of Clinical Pathology, National Liver Institute, Menoufiya University, Shebin El-Kom 32511, Egypt; 5 Genetic Engineering and Biotechnology Division, Microbial Biotechnology Department (Biomedical Technology Group), National Research Centre, Dokki 12622, Egypt; ashraftabll@yahoo.com; 6 Department of Immunology, Egypt Center for Research and Regenerative Medicine (ECRRM), Cairo 11517, Egypt; 7 Department of Pathology, National Institute of Infectious Diseases, Tokyo 208-0011, Japan; inok@nih.go.jp (N.I.-Y.); nnagata@nih.go.jp (N.N.)

**Keywords:** MERS-COV, egg yolk antibodies, IgY against MERS-COV, vaccine, passive immunotherapy, controlling of MERS-COV infection

## Abstract

The Middle East respiratory syndrome coronavirus (MERS-CoV) was identified in 2012 and causes severe and often fatal acute respiratory illness in humans. No approved prophylactic and therapeutic interventions are currently available. In this study, we have developed egg yolk antibodies (immunoglobulin Y (IgY)) specific for MERS-CoV spike protein (S1) in order to evaluate their neutralizing efficiency against MERS-CoV infection. S1-specific immunoglobulins were produced by injecting chickens with purified recombinant S1 protein of MERS-CoV at a high titer (5.7 mg/mL egg yolk) at week 7 post immunization. Western blotting and immune-dot blot assays demonstrated that the IgY antibody specifically bound to the MERS-CoV S1 protein. Anti-S1 antibodies were also able to recognize MERS-COV inside cells, as demonstrated by an immunofluorescence assay. Plaque reduction and microneutralization assays showed the neutralization of MERS-COV in Vero cells by anti-S1 IgY antibodies and non-significantly reduced virus titers in the lungs of MERS-CoV-infected mice during early infection, with a nonsignificant decrease in weight loss. However, a statistically significant (*p* = 0.0196) quantitative reduction in viral antigen expression and marked reduction in inflammation were observed in lung tissue. Collectively, our data suggest that the anti-MERS-CoV S1 IgY could serve as a potential candidate for the passive treatment of MERS-CoV infection.

## 1. Introduction

Respiratory infections involve a group of diseases affecting millions of people around the world, imposing a higher risk for elderly individuals and children [1]. MERS-CoV is an evolving zoonotic virus causing extremely deadly respiratory disease in human beings [2]. Middle East respiratory syndrome coronavirus (MERS-CoV) was first identified in 2012 [3,4]. As of January 2020, the incidence rate in humans has reached 2519 cases with an estimated death of 866 people, accounting for a 35% mortality rate [5]. Although most of the cases were reported in the Middle East, the virus has the potential to cause a global pandemic and spread through air travel, as in the case of the outbreak that took place in South Korea where more than 100 cases were reported [6]. The virus can be transmitted between dromedary camels, from camels to humans, and from humans to humans [7,8,9,10]. High risk groups, such as camel workers and healthcare workers, are at higher risk of encountering the infection unless precautionary measures are in place [11,12,13,14]. MERS-CoV continues to infect humans and is listed by the World Health Organization (WHO) and the Coalition for Epidemic Preparedness Innovations (CEPI) as a priority pathogen with the potential to cause a pandemic [15]. There are no approved treatments or vaccines available for MERS-CoV in humans or in camels. [15,16]. Therefore, it is crucial to implement strategies which will help in containing the spread of the infection [17].

Currently, anti-MERS-CoV neutralizing antibodies are being considered as a favorable treatment option for MERS-COV. Human monoclonal antibodies derived from recovered patients have been shown by several groups to neutralize and protect against MERS-CoV in infected mice [2,18,19,20]. Monoclonal antibodies interact with a single epitope on the MERS-CoV spike (S) protein which is subject to mutations that might lead to genetic alterations, increasing the chances of antibody escape [18,21]. Previously [22], passive immunotherapy using camel serum was shown to be a good therapeutic option and was reported to decrease the viral load and accelerate MERS-CoV clearance from the lungs of infected mice. Equine IgG-derived F(ab’)2 fragment showed comparable results when administered to MERS-CoV infected mice [23]. An egg yolk has a single class of antibody (IgY) as compared to mammalian serum, and hence it is easily isolated via precipitation techniques [24]. The immunoglobulin Y antibodies (IgY-Abs) are the primary immunoglobulins in oviparous animals, specifically equivalent mammalian IgG, which is transferred to the egg yolk [25]. Over the past few years, IgYs have been the focus of several studies providing a potential alternative approach for passive immunization and the prevention of several infectious diseases [26]. IgYs are less harmful than IgGs because they do not have the capability to bind to a human Fc receptor or react with mammalian complement system, thus they do not generate undesired immune reactions [27]. IgY antibodies can be produced on a large scale, providing a non-invasive, animal friendly, efficient, and cost-effective alternative for immunotherapeutics [28,29,30,31,32]. Furthermore, the binding avidity of the IgY antibodies to target antigens is more than mammalian IgG antibodies [33] and can be easily produced against conserved mammalian proteins as compared to IgG antibodies produced in other mammals, because of the evolutionary distance present between mammals and birds [24].

Specific IgY antibodies have been successfully developed and proven to be highly effective for preventing and treating respiratory diseases caused by bacteria and viruses, such as influenza A virus [34,35,36,37], influenza B virus [38], SARS-CoV [39], bovine respiratory syncytial virus [40], and *Mycobacterium tuberculosis* [41]. IgY technology has already been successfully applied in clinical trials against *Pseudomonas aeruginosa* lung infection [42], and anti-*P. aeruginosa* IgY has been approved by the Swedish Medical Products Agency for treating cystic fibrosis patients. In 2008, the European Medicines Agency granted an orphan drug designation to IgY antibodies for the treatment of *P. aeruginosa* infections associated with cystic fibrosis [43]. A recent study demonstrated that IgY antibodies transiently decreased *P. aeruginosa* colonization of the airway in mechanically ventilated piglets [44]. Moreover, specific IgY antibodies could protect mice against pneumonia caused by *Acinetobacter baumannii* [43].

The MERS-CoV S protein consists of S1 and S2 subunits [45]. The MERS-CoV S1 subunit has been used as a potential vaccine target, and several vaccines have been constructed using this region [45]. DNA-based MERS-CoV S1 vaccines were found to elicit antibody and cellular immune responses and neutralizing antibodies that confer protection against MERS-CoV infection in a mouse model [46,47]. In the current report, we describe the production and neutralizing activity of anti-MERS-CoV S1 IgY antibodies.

## 2. Materials and Methods

### 2.1. Immunization of Laying Hens

Eight Lohmann laying hens (25 weeks old) were purchased from a local broiler farm (Algharbia Breeding Company, Jeddah, Saudi Arabia) and used for egg production. Animals were placed in cages dedicated to broiler chickens in groups of two animals per cage, in a regimen of light-dark cycle (12:12 h), at room temperature around 24 ± 3 °C. Water and food for commercial laying hens were offered ad libitum. Four chickens (immunization group) were immunized by injection of 200 µg of recombinant MERS-CoV spike protein (S1) (Sino Biological Inc, Beijing, China, Cat: 40069-V08H) in the left and right side of pectoral muscle at days 0, 14, 28, and 49. The recombinant protein was emulsified at a ratio of 1:1 in complete Freund’s adjuvant (Sigma, USA, Cat: F5881) for the first immunization, and incomplete Freund’s adjuvant (Sigma, USA, Cat: F5506) was similarly used for the following booster immunizations. The suspension was mixed by pipetting up and down in a 19-gauge needle attached to a 5-mL syringe until the emulsion was stable. The control group (*n* = 4) was injected with phosphate-buffered saline (PBS) and the corresponding adjuvant. Blood samples for determining antibody response were taken from the birds before each immunization and on the last day before slaughter. Eggs were collected daily 1 week before and 24 h after initial immunization and continued for 12 weeks. The eggs were stored at 4 °C until IgY isolation from the yolks.

### 2.2. Isolation and Purification of Yolk IgY

Egg yolks for each hen of the two groups (immunized and nonimmunized, 4 hens per group) were separated from egg whites, washed with deionized water and the yolks of each week of the individual hen were pooled to represent the IgY-Ab for this week. The purification of IgY antibodies was performed using Pierce Chicken IgY purification kit (Thermo Fisher Scientific, Waltham, MA, USA, Cat: 44922) according to the manufacturer’s instructions. Egg yolks were separated from the egg white using the Egg Separator, egg sacs were rolled onto a clean, dry paper towel to remove remaining egg white. The egg sacs were punctured with the Pasteur pipette and the egg yolks were collected. Egg yolks were mixed with five volumes of Delipidation Reagent with gentle continuous mixing. The sample mixtures were centrifuged for 15 min at 10,000× *g* in a refrigerated centrifuge to remove the precipitated debris. While stirring gently, an equal volume of cold IgY Precipitation Reagent was added to the supernatant, and the mixture was centrifuged again after incubation for 1 h at 4 °C. PBS (equal to the original volume of the egg yolk) was added to the precipitated pellets that contained IgY and mixed gently until it is completely dissolved filtered through a 0.45-µm filter and stored at −20 °C. The IgY concentration was determined using a NanoDrop 2000 spectrophotometer system (Thermo Scientific, USA).

### 2.3. Sodium Dodecyl Sulfate–Polyacrylamide Gel Electrophoresis

Sodium dodecyl sulfate–polyacrylamide gel electrophoresis (SDS-PAGE) was performed to determine the purity and molecular weight of IgY. A 12% polyacrylamide gel was used with a Mini-PROTEAN^®^ 3 cell (Bio-Rad Laboratories, Hercules, CA, USA, Cat: 165-3301). The analysis was conducted under reducing conditions. The sample was mixed with 2× sample buffer and boiled for 10 min at 100 °C. A total of 25 μL of purified IgY was loaded into each well. A pre-stained blue protein marker (MOLEQULE-ON, New Zealand) was used as a molecular weight marker. Electrophoresis was performed at room temperature in running buffer (Tris-glycine buffer) at 200 V for 40 min. The protein bands were visualized with Coomassie Brilliant Blue stain and analyzed using GeneTools image analysis software (Syngene, Cambridge, UK).

### 2.4. Reactivity of Anti-S1 IgY Antibodies

The reactivity and titer of the generated anti-S1 IgY antibodies was determined by ELISA. Briefly, 96 wells microtiter plates coated with MERS-CoV-S1 antigen (Sino Biological Inc., China) at 500 ng/mL in PBS (0.01 M, pH 7.4) (100 µL/well) and kept at 4 °C overnight. Coated plates were washed three times with wash buffer (1 × PBS, tween-20) followed by blocking with 250 μL of blocking buffer (5% skim milk in PBS-Tween) at room temperature for an hour, washed 3 times with washing buffer. Serial dilution of the IgY antibodies were used to determine the titer in egg yolk and serum from immunized and non-immunized hens, starting from 1:50 in blocking buffer (each dilution was tested in triplicate) and the plate was incubated at 37 °C for 1 h. A 1:10,000 dilution of HRP (horseradish peroxidase)-conjugated rabbit anti-chicken IgY (Abcam, Cambridge, UK, Cat: ab 6753) was added to each well (100 μL/well) and incubated for 1 h at 37 °C. The plates were washed, and color was developed by adding TMB 100 μL/well substrate solution (Promega, Madison, WIS, USA, Cat: G7431) and incubated for 30 min. Finally, the reaction was stopped by adding 100 μL/well of 2 M H_2_SO_4_. The OD was measured at 450 nm using a microtiter plate reader (ELX800 Biokit) using PBS as a blank, and purified IgY from non-immunized hens serving as a negative control. A titer of anti-S1 IgY was evaluated as the maximum dilution of the sample, which resulted in an O.D value of 2.1 times than that of the negative control. 

### 2.5. Western Blot Assay

In order to determine the specificity of the anti- MERS-CoV S1 IgY antibodies, western blotting was implemented as previously described with some modifications [48]. The recombinant S1 protein (500 ng) was mixed with 20 µL of electrophoresis sample buffer and subjected to SDS–PAGE in a 14% slab polyacrylamide gel separated by a 4% stacking gel at 200 V for 40 min at RT. The gel and blotting papers were equilibrated in transfer buffer for 10 min. The S1 protein was then mobilized electrically onto Polyvinylidene fluoride (PVDF) membrane activated by methanol (Thermo Fisher, USA) at 30 V for overnight. The PVDF membrane was cut into 0.5-cm strips, which were blocked with Tris-buffered saline containing 0.1% Tween 20 (TBS-T) and 5% skimmed milk for 1 h at RT, washed 3 times for 10 min, followed by incubating the membrane with a 1:50 dilution of anti MERS S1 IgY antibodies. The strips were then washed 3 times with TBS-T for 10 min and incubated with HRP conjugated Rabbit Anti-Chicken IgY H&L (Abcam, UK) at 1:10,000 dilution in blocking buffer, for 1 h at RT. Then, the strips were washed three times for 10 min. After washing, the strips were incubated with HRP colorimetric substrate (Immun-Blot Opti-4CN colorimetric Kit, Bio-Rad) for 15 min at RT. Finally, the reaction was ceased by rinsing the strips with distilled water. The strips were photographed after the development of visible bands.

### 2.6. Immuno-Dot-Blot Assay

A dot-blot assay was performed to determine the specificity of the purified anti-S IgY antibodies. The PVDF membrane was activated by soaking in methanol for 15 s, then washed with distilled water. Three different concentrations (500, 100, and 50 ng) of each recombinant antigen (S, S1, nucleocapsid protein [NP], and receptor binding domain (RBD)) was separately dot-blotted onto PVDF membrane. The membrane was incubated in 20 mL of blocking buffer for 1 h at room temperature. After being washed three times with TBS-T, the PVDF membrane was immersed in primary antibody, anti-S IgY antibodies of the MERS-COV (at 1:200 dilution), in blocking buffer with gentle agitation for 1 h at room temperature. The membrane was next incubated with HRP-conjugated rabbit anti-chicken IgY (Sigma, USA) as a secondary antibody (at dilution 1:10,000) in blocking buffer with gentle agitation for 1 h at room temperature. After being washed, the membrane was incubated with HRP colorimetric substrate (Immun-Blot Opti-4CN colorimetric Kit, Cat. No. 1708235) to 30 min at room temperature. The reaction was stopped with distilled water, and the image was captured after dot color development.

### 2.7. Immunofluorescence Assay

To perform the immunofluorescence assay, Vero E6 cells (ATCC, Cat number CRL-1586™) were inoculated with MERS-CoV and the infected cells were harvested after 48 h of infection. The cells were collected by centrifugation at 1500 rpm for 5 min and washed twice with wash buffer consisting of 1 mL of BD Perm/Wash buffer (BD Biosciences, San Diego, CA, USA, Cat: 554723). One hundred microliters of the cell suspension were added to tubes containing 200 μL of blocking buffer (BD Cytofix/Cytoperm solution, Cat: 554714) and incubated for 1 h at room temperature. Cells were then washed twice with wash buffer.

The IgY antibodies were diluted to 570 μg/mL in Dulbecco’s modified Eagle’s medium (DMEM) supplemented with 2% fetal bovine serum (FBS). Two hundred microliters of the diluted anti-S1 IgY antibodies was added to each tube containing infected cells, followed by incubation for 1 h at room temperature. Cells were washed twice with wash buffer, and 100 μL of fluorescein isothiocyanate (FITC)-conjugated anti-chicken antibodies (Sigma, USA, Cat: F4137) was added (in a 1:2500 dilution) and incubated for 1 h at room temperature. Cells were washed twice with wash buffer, 30 μL of cell suspension was fixed on a slide and observed under fluorescent microscope, and images were captured.

### 2.8. Neutralization Assay

Live virus experiments were performed in biosafety level 3 laboratory of the Special Infections agent unit, King Fahd Medical Research Center, King Abdulaziz University in Jeddah. Neutralizing assay was performed as previously described [49]. Briefly, MERS-CoV isolate at MOI of 0.01 (500 μL) in the presence or absence of IgY antibodies were added to an equal volume of serial dilutions of the IgY antibodies for 1 h. The mixture was then inoculated in triplicate onto Vero E6 cells (10,000 cells/well) in 96-well plates in viral inoculation medium (DMEM with 2% FBS, 1% penicillin/streptomycin, and 10 mmol/L HEPES (pH 7.2)). Cells were then incubated in a humidified 5% CO_2_ incubator at 37 °C for 2–3 days or until 80–90% cytopathic effect (CPE) was reached in positive virus control wells (Virus with no added IgY-Abs). The IC100 neutralization of the antibody was determined as the reciprocal of the highest dilution at which no CPE was observed.

### 2.9. Plaque Reduction Neutralization Test

Plaque reduction neutralization assay was performed according to Landry et al. [49] as performed to evaluate the neutralizing activity of anti-S1 IgY antibodies in MERS-CoV. Serial dilutions of the IgY antibodies were incubated with an equal volume of 0.01 MOI MERS-COV at 37 °C for 30 min. Subsequently, 200 µLs of the incubated mixture were added to 95–100% confluent Vero E6 cells in 12-well plates. Each assay also included, a cell control (PBS and cells) and a virus control (virus and cells). After 2 h of incubation at 37 °C, the surface of the Vero cells was covered with agarose-containing overlay medium of 1.5 mL to control the indiscriminate spreading of the virus. Plates were incubated for 72 h at 37 °C in a 5% carbon dioxide atmosphere. Vero cells were fixed with 10% formalin in phosphate-buffered saline followed by staining with 1% crystal violet in 50% ethanol. The 50% inhibitory concentration (IC50) of specific IgY against MERS-COV virus was evaluated via the Reed–Muench method [50].

### 2.10. Effect of Anti-S1 IgY Antibodies in Transgenic Mice after MERS-CoV Infection

A mouse model of MERS-CoV was used in this study according to [51]. Briefly, transgenic (Tg) mice on a C57BL/6NCr (SLC Inc., Hamamatsu, Japan) background were developed; these mice expressed human CD26/dipeptidyl peptidase 4 (hDPP4), a functional receptor for MERS-CoV, under the control of an endogenous hDPP4 promoter. hDPP4-Tg mice were intranasally inoculated with MERS-CoV: HCoV-EMC 2012 strain, kindly provided by Dr. Bart Haagmans and Dr. Ron Fochier (Erasmus Medical Center, Rotterdam, the Netherlands), at 10^6^ TCID_50_ (*n* = 10–12). Mice were then injected peritoneally with 500 μg of anti-S1 IgY antibodies at 6 h and 1 day post infection. Body weight was observed for 8 days post infection. Animals were sacrificed at 1, 3, and 5 days post infection p.i. (*n* = 4), and lung tissues were collected for virological detection. After the observation period (8 days), the remaining mice were sacrificed for histopathological evaluations. All work with MERS-CoV infection and passive immunization of mice was conducted at the National Institute of Infectious Diseases, Tokyo, Japan. Stocks of MERS-CoV were propagated and titrated on Vero E6 cells and cryopreserved at −80 °C. Viral infectivity titers are expressed as the TCID_50_/mL on Vero E6 cells and were calculated according to the Behrens-Kärber method. Work with infectious MERS-CoV was performed under biosafety level 3 conditions.

### 2.11. Histopathology and Immunohistochemistry

Lung tissues were obtained after anesthetizing the mice and perfusion with 2 mL of 10% phosphate-buffered formalin, lungs were then harvested and fixed. Fixed tissues samples were embedded in paraffin, sectioned, and subjected to hematoxylin and eosin staining. Formalin-fixed paraffin-embedded tissue sections were autoclaved at 121 °C for 10 min in retrieval solution at pH 6.0 (Nichirei Biosciences Inc., Tokyo, Japan) for antigen retrieval in preparation for immunohistochemistry (IHC). MERS-CoV antigens were detected utilizing a polymer-based detection system (Nichirei-Histofine Simple Stain Mouse MAX PO(R); Nichirei) with a rabbit anti-MERS-CoV nucleocapsid antibody (40068-RP01; Sino Biological Inc., Beijing, China). Peroxidase activity was detected with 3,3′-diaminobenzidine (Sigma-Aldrich), and hematoxylin was used for counterstaining.

### 2.12. Quantitative Analysis of Inflammation and Viral Antigen Positive Cells

The inflammation area was assessed by HE staining on 3-µm-thick paraffin embedded sections from Tg mice at 8 days p.i. Light microscopic images were obtained with a DP71 digital camera and cellSens software (Olympus Corporation, Tokyo, Japan). The images were taken under low-power magnification. Inflammation was evaluated by measure of area for each section of the three lobes (average of section area: 3.343 ± 2.615 mm^2^) collected from each Tg mouse. The inflammation areas were traced with the contour measurement program of Neurolucida (version 12, 64 bit; MBF Bioscience, Williston, VT, USA) and analyzed with Neurolucida Explorer (MBF Bioscience).

Viral antigen was detected by IHC on the continuous section from the paraffin embedded sections. Viral antigen positive cells were counted using the images under high-power magnification (observation area: 0.147 mm^2^).

### 2.13. Statistical Analysis

Data are expressed as the means with standard errors. Statistical analyses were performed using Graph Pad Prism 8 software (GraphPad Software Inc., La Jolla, CA, USA). Intergroup comparisons (virus titers in the lung and body weight curve) were performed using analyses of variance (two-way ANOVAs) followed by Bonferroni’s multiple comparisons test. Two group comparisons (quantitative analysis of inflammation and viral antigen positive cells) were performed using analyses of the Mann–Whitney test. A *p* value of <0.05 was considered statistically significant.

### 2.14. Ethics Statement

The experimental protocol for immunization and handling of chicken was reviewed and approved by the Unit of Biomedical Ethics Research Committee, Faculty of Medicine, King Abdulaziz University (Permit No: 120–18). 

Experiments performed using recombinant DNA and pathogens were approved by the Committee for Experiments Using Recombinant DNA and Pathogens at the National Institute of Infectious Diseases, Tokyo, Japan. Animal studies were performed strictly following the Guidelines for Proper Conduct of Animal Experiments of the Science Council of Japan. Animal experiments were performed in strict compliance with animal husbandry and welfare regulations. All animals were housed in a Japan Health Sciences Foundation-certified facility. Animal experiments were approved by the Committee on Experimental Animals at the National Institute of Infectious Diseases in Japan, and all experimental animals were handled in accordance with biosafety level 3 animal facilities according to the committee guidelines.

## 3. Results

### 3.1. Isolation and Purification of IgY

**Figure 1:**
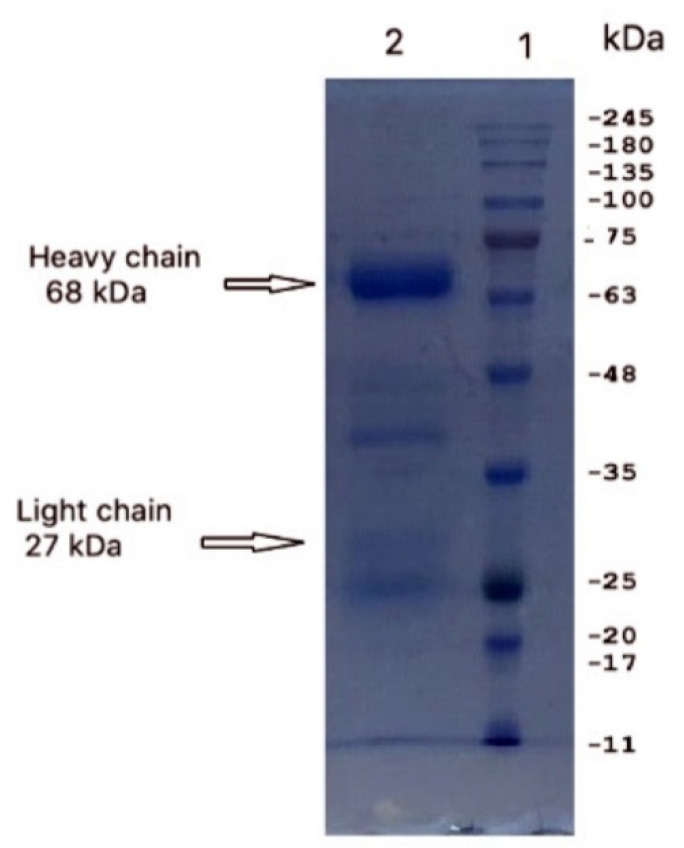
IgY purified from chicken egg yolk was resolved on 12% SDS-PAGE gels and visualized by staining with Coomassie Brilliant Blue. Two major protein bands with molecular weights of 68 kDa and 27 kDa, representing the heavy and light chains of IgY, respectively, were detected (arrows).

SDS-PAGE revealed that the IgY preparation dissociated into two protein bands, a major band at ~68 kDa (heavy chain) and a minor band at ~27 kDa (light chain) with 90% purity (Figure 1). The total IgY contained in 1 mL of egg yolk was estimated to be 5.7 mg. Each egg yolk was approximately 15 mL, indicating that approximately 85.5 mg of total IgY could be obtained from a single egg.

### 3.2. Dynamics of Anti-S IgY Antibodies in Chickens’ Sera and Egg Yolks

**Figure 2:**
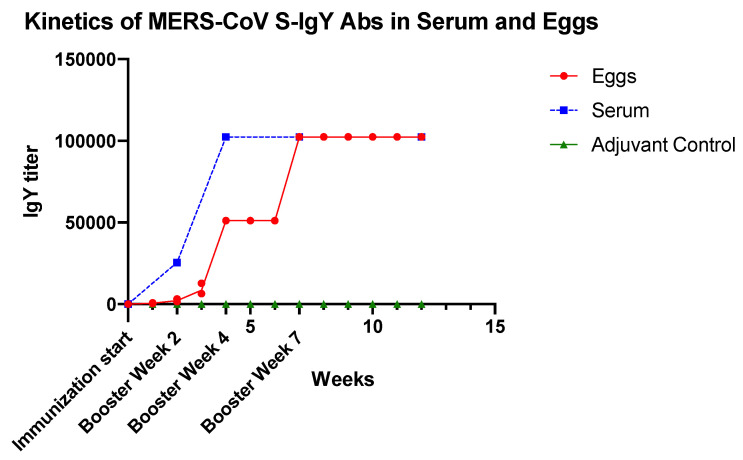
Kinetics of serum and egg yolk anti-MERS COV-S IgY antibodies response of chickens after immunization with MERS COV-S recombinant protein compared with the adjuvant-immunized chicken (adjuvant control). Each week is represented by a pool of egg yolks of individual chicken in each group (S1-immunized and adjuvant-immunized).

A steady increase in serum MERS-CoV S1-specific IgY titers was observed after the first immunization, reached a peak in week 7, and remained high until week 12. Sera of chickens immunized with PBS-adjuvant control did not show any reactivity to MERS-CoV-S. The anti-MERS-CoV S antibody titers in the eggs were not detected until the third week after immunization then started rising until reaching a peak in week 7. They plateaued at this level till week 12 (Figure 2).

### 3.3. Immunoreactivity of Anti-S1 IgY of the MERS-COV

**Figure 3:**
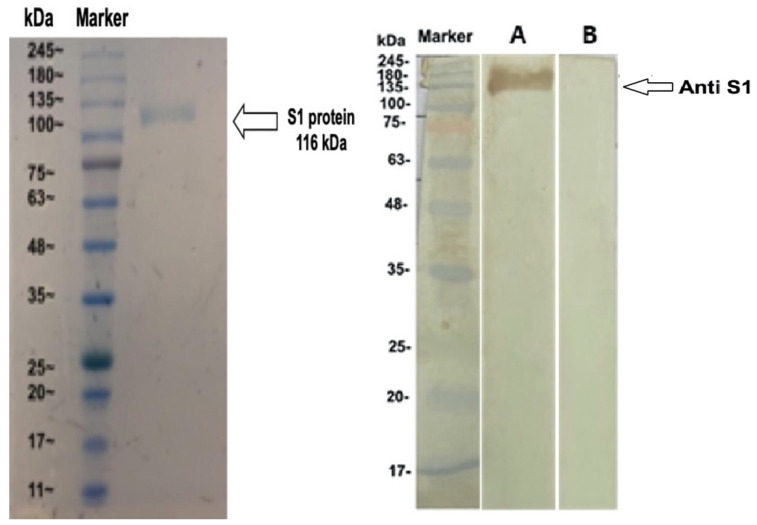
Western blot analysis of anti-MERS-COV rS1 IgY antibodies. (Left) The S1 protein of MERS-COV was subjected to SDS-PAGE under reducing conditions; (Right) Western blot analysis of the anti-S1 IgY antibody response. SDS gels were electrically transferred onto nitrocellulose membranes and probed with IgY from immunized and nonimmunized hens (marker: molecular maker; lane A: S1-immunized IgY; lane B: adjuvant-immunized IgY). The strips were processed separately and pasted beside each other for documentation.

The specificities of anti-MERS-CoV S1-IgY antibodies were also tested by Western blot analysis. The IgY induced by MERS-CoV S1 was able to recognize the S protein at approximately 116 kDa (Figure 3).

### 3.4. Dot Blotting

**Figure 4:**
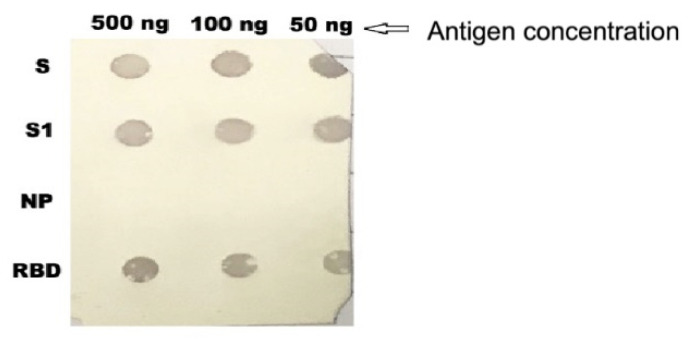
Dot blotting analysis. Purified anti-S1 IgY antibodies showed reactivity with different concentrations of the spike protein (S), S1, and receptor binding domain (RBD), but had no reactivity with nucleocapsid (NP) protein of MERS CoV.

The specificities of anti-S1 IgY antibodies were also confirmed by dot immune- blot assay. Purified IgY antibodies showed reactivity with S, S1, and RBD, while no reactivity was observed with MERS CoV NP (Figure 4).

### 3.5. Intracellular Immunofluorescent Detection of IgY Antibodies Binding to MERS-CoV

**Figure 5:**
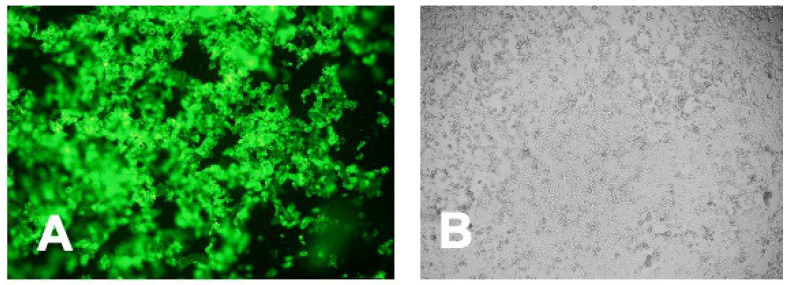
Recognition by anti-S1 IgY antibodies of viral antigen expressed in MERS-CoV-infected Vero E6 cells, using indirect immunofluorescence assay. (**A**) Vero E6 cells inoculated with MERS-CoV and stained with anti-S1 IgY antibodies and FITC-conjugated anti-chicken antibodies; and (**B**) control adjuvant IgY (Bright-field).

The intracellular binding of the generated IgY antibodies was confirmed by immunofluorescent staining of the IgY antibodies using FITC-labeled anti-chicken antibodies. Figure 5A shows the cytoplasmic fluorescence of the treated cells indicating the binding of the IgY antibodies to the viral antigen inside the cells. Cells with adjuvant IgY antibodies showed no fluorescence (Figure 5B).

### 3.6. Anti-S1 IgY Neutralizes MERS-CoV Infection

**Figure 6:**
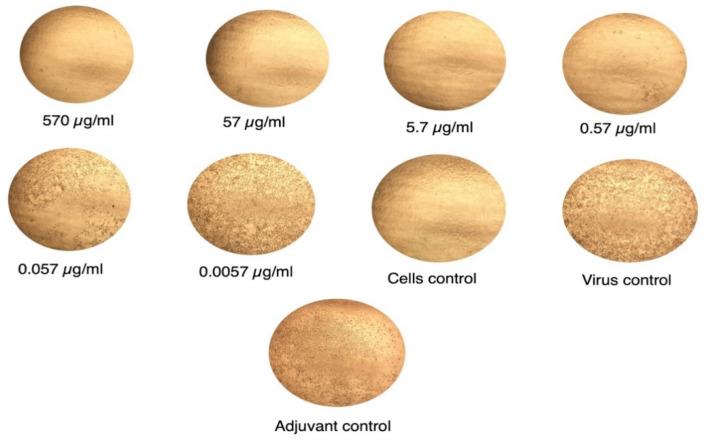
Examples of different concentrations of anti-S1 IgY antibodies tested against MERS-CoV on Vero-E6 cells examined by CPE. The IC100 neutralization of the antibody were determined as the reciprocal of the highest dilution at which no CPE was observed.

Anti-S1 IgY could potently neutralize infection of live MERS-CoV in permissive Vero cells with 100% neutralization at concentration <31.2 µg/mL, whereas nonspecific antibodies from adjuvant-immunized chickens did not exhibit antiviral activity against MERS-CoV infection up to 1 mg/mL (Figure 6). These results suggest that anti-S1 MERS-CoV IgY antibodies exhibited a potent ability to neutralize MERS-CoV infection. Inhibitory concentration of 100% (IC100) was determined as the IgY dilution at which no CPE was observed in the cells.

### 3.7. IgY Inhibits Virus Replication In Vitro

**Figure 7:**
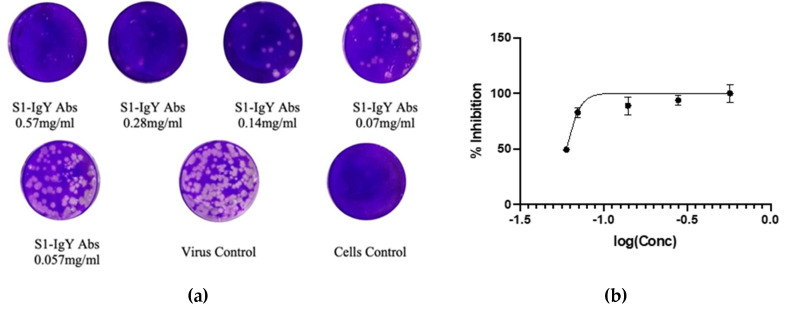
Evaluation of the neutralizing potential of anti-S1 IgY antibodies, using plaque reduction neutralization test. (**A**) MERS-CoV (MOI 0.01) was incubated with different concentrations of anti-S1 IgY antibodies and added to Vero E6 cells. After virus adsorption, agar medium was added to the Vero E6 cells, and the plaques that formed were stained with crystal violet, each IgY concentration was tested in triplicate. (**B**) Percent inhibition of anti-S1 IgY antibodies with different concentrations. The best fit equation is:

To further confirm the antiviral activity observed with the above neutralization assay, a plaque reduction assay was performed on Vero E6 cells inoculated with MERS-CoV pre-incubated with IgY antibodies. As shown in Figure 7, the specific IgY significantly (*p* < 0.005, IgY concentration at 2 mg/mL compared with virus control) inhibited MERS-CoV virus replication in Vero cells with an IC50 of 0.06 mg/mL.

Y = 100/(1 + 10^((LogIC50-X)*HillSlope)).(1)

### 3.8. IgY Confers In Vivo Protection in Virus-Challenged Mice

**Figure 8:**
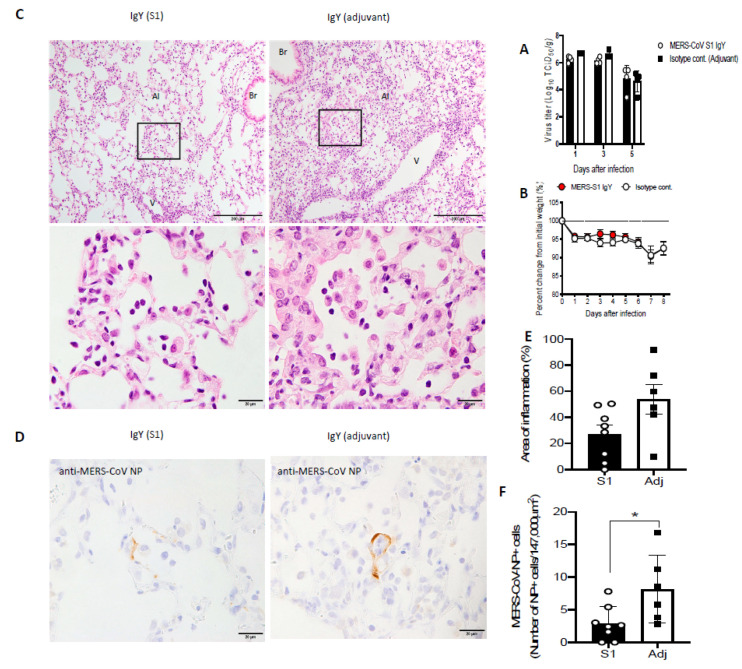
(**A**) Viral titer in the lungs of MERS-CoV mice treated with anti-SI IgY antibodies and control IgY (adjuvant). (**B**) Body weight changes after MERS-CoV infection between anti-SI IgY antibodies and IgY of adjuvant control group. (**C**–**F**) Histopathology of the lungs from human dipeptidyl peptidase 4 (hDPP4)-transgenic mice on day 8 after inoculation with MERS-CoV. Representative histopathological findings of mice with the highest cellular infiltration in alveoli by H&E staining (**C**) Massive mononuclear cell infiltrations including macrophages and lymphocytes with regenerated type II pneumocytes were seen in adjuvant control group (right column), but less in the anti-S1 IgY treated group (left column). Scale bars: 200 μm (upper row) and 20 μm (lower row). Al, alveoli; Br, bronchi; V, vessel. Detection of viral antigen in lung tissues of mice by immunohistochemistry (**D**) A few antigen positive cells were seen in the lungs of anti-S1 IgY treated group compared to adjuvant control group. Quantification of inflammation areas (**E**) The area of pulmonary lesion was determined based on the mean percentage of affected areas in each section of the collected lobes form each animal (*n* = 8 or 6). Circles indicate averages from three observation lobes in each mouse. *p* = 0.1709 by Mann-Whitney test. Numbers of viral antigen positive cells in the alveoli (**F**) Data were obtained from 8 or 6 mice. Circles indicate averages of 5 observation fields in each mouse. * *p* = 0.0196 by Mann-Whitney test.

Next, we determined the effect of anti-S1 IgY treatment in vivo. Intraperitoneal treatment with anti-S1 IgY antibodies resulted in no significant difference in the viral titer in the lung compared with the control adjuvant IgY, but the titer was slightly lower at day 3 post infection in the anti-S1 IgY group compared with the control group (Figure 8A).

Body weight changes were not significantly different after intranasal inoculation with 10^6^ TCID_50_ of MERS-CoV in hDPP4-Tg mice between MERS-CoV S1 IgY antibodies and control adjuvant IgY groups (Figure 8B). Histopathological investigations revealed that Tg mice showed progressive pulmonary inflammation associated with acute viral infection on day 8 post infection. Inflammatory reactions, including partial and/or mild cellular infiltration with mononuclear cells and macrophages in response to viral infections, were observed in alveolar areas of lung tissue (Figure 8C), but interestingly, the group treated with anti-S1 IgY antibodies showed a markedly decreased inflammatory reaction compared with the control group (Figure 8C,E). Moreover, IHC using an anti-MERS-CoV NP polyclonal antibody in lung tissues revealed a significant reduction of the viral antigen-positive cells in the lungs of the anti-S1 IgY-treated group (Figure 8D) compared with the adjuvant IgY control group, *p* = 0.0196 (Figure 8F).

## 4. Discussion

The spread of MERS-CoV infections in humans from dromedary camel reservoir, high rate of mortality and easy human-to-human transmission is a serious public health threat. The absence of approved vaccines and treatments urges the need for effective preventive strategies [52].

Targeting critical viral entry glycoproteins using antibody therapies is increasingly identified as a promising antiviral strategies for protecting humans from lethal infections [53]. For MERS-CoV, passive immunization studies with neutralizing antibodies in small animals support the idea that antibody therapies may provide a promising approach in the fight against MERS-CoV infection [2]. IgY antibodies has gained great interest in the fight against infectious diseases as they provide a passive, economical, convenient and highly productive source of antibodies [28]. Previous studies have shown that passive immunity using IgY is efficient in protecting against bacterial or viral respiratory infection [54]. Avian IgY and egg powder have been declared as safe, by the United States Code of Federal Regulation [55]. IgY antibodies has certain advantages over mammalian serum immunoglobulins in terms of productivity, animal welfare and specificity. It also shows a greater avidity to conserved mammalian proteins [56]. Nguyen et al. (2010) demonstrated that a hen typically lays 280 eggs per year and each egg yolk generally contains 150–200 mg of IgY, which has 2–10% antigen-specific antibodies [34]. Thus, if each yolk contains 200 mg of IgY, up to 20 mg will be the IgY of interest [57].

In the present study, specific IgY antibodies were developed against MERS-COV S1 protein. This study utilized the spike protein S1 of the MERS-COV as an immunogen to inoculate hens, thereby producing anti-MERS S1 IgY-Abs. The S1 subunit contains the RBD, which binds to the host receptor DPP4 (also known as CD26) and stimulate specific neutralizing antibodies production [58]. Various studies have confirmed the role of the RBD in the development of protective immunity against MERS-CoV infection [59,60,61].

After immunization with a small dose of antigen, the chicken can continuously produce eggs containing antigen-specific IgY antibodies [28]. This study detected a continual rise of IgY titer in the egg yolk from the third week post first immunization, which peaked at the seventh week and plateaued at a high level until the end of experiment (12 weeks). This rapid increase in antibody titer indicates a robust immune response in immunized hens, as reported in other studies [62]. Similar results were observed by Wallach et al. [36] and they found a long-lasting humoral immune response for a minimum of two months after immunization with different strains of influenza viruses (H1N1, H3N2, and H5N1), and additional boosts were not needed. Other studies revealed that hens maintain a high antibody titer against a variety of antigens used for immunization for up to 3–4 months [63].

S1 Protein promotes binding to host cell surface molecules during the phase of viral attachment [64]. S1 is a domain of MERS-COV full length spike protein [65] and the receptor binding domain (RBD) is a fragment located in the middle of S1 [66]. In the Western blot analysis, the anti-S1 IgY antibodies exhibited immunoactivity against S1 virus protein. This reactivity was also observed when anti-S1 virus IgY antibodies binds to S and RBD proteins. However, it did not show any reactivity with the recombinant nucleocapsid protein in a dot-blot immunoassay, confirming that the anti S1 IgY antibodies are antigen specific. This binding was observed not only to the recombinant antigens but was also found to bind intracellularly to whole virus as shown in the immunofluorescent assay on whole virus infected cells, indicating the potential ability of the anti-S1 IgY antibodies to bind and neutralize MERS-CoV in vitro. The increased antigenic recognition of IgY are attributed to the unique antibody maturation process. [67,68,69]. The IgY antibodies developed in this study showed immunoaffinity for the viral S1 protein and had an effective in vitro neutralizing activity and in vivo therapeutic efficacy. The difference in the inhibitory concentration values between the plaque test and the microneutralization assay may be related to different techniques used in the two methodologies.

Because wild-type mice are not permissive to MERS-CoV infection [70], the passive immunotherapeutic effect of anti-IgY antibodies was evaluated in the hDPP4-Tg mouse model [71]. One of the indicators for the effect of the anti-S1 IgY-antibodies is reduced weight loss in infected animals. In the current study, there was no significant difference in body weight between the treatment group and the controls. This lack of difference might be attributable to the short time duration of the study, as reported by New et al. [72]. Furthermore, Yoshikawa et al. [51] found that both young and adult Tg mice infected with MERS-CoV showed transient weight loss, suggesting that body weight is not a useful parameter for the assessment of the efficacy of anti-S1 IgY antibodies. The virus titers in the lungs of anti-S1 IgY-treated mice at day 3 after infection were slightly reduced compared with mice receiving nonspecific IgY antibodies, as reported by earlier studies defining the optimum time for assessing the efficacy of anti-MERS-CoV agents [20]. A significant decrease in the viral titer in the lungs of infected mice was observed after three days. A decrease in viral titer by five days post infection was reported for both treatment and control groups in this animal model by Iwata-Tishikawa et al. [51]. Histopathologic examination and immunostaining (e.g., IHC) of lung tissues are essential to better understand disease pathogenesis and evaluate novel treatments of MERS and SARS-COV and current (and future) virus outbreaks [73,74,75,76]. Interestingly, the anti-S1 IgY antibody-treated group showed a statistically significant (*p* = 0.0196 by Mann–Whitney) decrease in viral antigen positive cells compared to the control group. The reason for the difference in neutralization activity observed in the lung tissue could be due to the different templates used for detection. Tissue culture infection dose TCID50 assay interpretation can be subjective and highly variable, a sufficient number of replicates must be performed to obtain statistically significant results [77,78], on other hand, the use of IHC has a particular advantage by allowing localization of virus within the lung tissue [79]. Recent advances in IHC with specific antibodies confer great advantages in relation to diagnosing viral infection with regard to both sensitivity and specificity [80]. Immunohistochemistry revealed that the viral antigen positive cells prolonged until eight days post infection. As the reviewers say, we think that a better effect can be obtained by increasing the antibody administration period. IHC result was confirmed by marked decrease of the quantified inflammatory cellular response in the S1-IgY treated group compared to the control group.

## 5. Conclusions

Taken together, our data provide evidence for the specific and efficient neutralization of MERS-CoV using anti-S1 IgY antibodies both in vitro and in a Tg animal model. The data presented here provide the first evidence for the potential use of the generated IgY antibodies as a therapeutic vaccine against MERS-CoV. After evaluation in clinical trials, anti-S1 IgY antibodies might be used for the treatment of MERS-CoV, especially in high-risk populations with immature or weakened immunity. Furthermore, the IgY antibodies can be used for the treatment of MERS-CoV in camels, which are the animal reservoir responsible for transmitting the virus to humans. The data generated in this study provide a platform for the generation of specific and efficient IgY antibodies against other coronaviruses in future studies.
